# Supplementary material for: Functional and structural analyses of IMP-27 metallo-β-lactamase: evolution of IMP-type enzymes to overcome Zn(II) deprivation
Source: Microbiol Spectr. 2024 Nov 7;12(12):e00391-24. doi: 10.1128/spectrum.00391-24 (PMC11619291; doi:10.1128/spectrum.00391-24)
Supplement: Supplemental material — Tables S1 to S4, and Figures S1 and S2. [file spectrum.00391-24-s0001.docx]

**Table S1** Data collection and structure refinement

| Data collection |  |
| --- | --- |
| X-ray source | PF BL-5A |
| Space group | *P* 2_1_2_1_2_1_ |
| Cell dimensions |  |
| *a*, *b*, *c* (Å) | 47.59, 71.25, 136.21 |
| α, β, γ (°) | 90.00, 90.00, 90.00 |
| Wavelength (Å) | 1.000 |
| Resolution (Å) | 68.11–1.70 (1.79–1.70) |
| Observed reflections | 365524 (53327) |
| Unique reflections | 51881 (7438) |
| *R*_merge_ | 0.091 (0.498) |
| *I* /σ (*I* ) | 13.4 (3.7) |
| Completeness (%) | 100.0 (100.0) |
| Refinement |  |
| Resolution range for refinement (Å) | 39.57–1.70 |
| Unique reflections | 49146 |
| Number of atoms | 3758 |
| Protein | 3428 |
| Water | 317 |
| Zinc ions | 4 |
| *R*_factor_ | 0.178 |
| *R*_free_ | 0.200 |
| Average B factor (Å^2^) |  |
| All atoms | 19.0 |
| Protein | 18.7 |
| Water | 23.8 |
| Ligands (Zn ion) | 15.1 |
| RMSD |  |
| Bond Length (Å) | 0.012 |
| Bond Angle (°) | 1.696 |
| Ramachandran plot (%) |  |
| Favored | 96.3 |
| Allowed | 2.5 |
| Outliers | 1.2 |

**TABLE S2** Comparison of Zn(II)-ligand distance in the IMP-type enzymes.

|  |  | Distance(Å) | | | | | |
| --- | --- | --- | --- | --- | --- | --- | --- |
|  |  | IMP-27  (6L3S)^*^ | IMP-1  (5Y5B)^*^ | IMP-2  (4UBQ)^*^ | IMP-6  (6LVJ)^*^ | IMP-13  (6R78)^*^ | IMP-18  (5B3R)^*^ |
| Zn1 – Zn2 |  | 3.5 | 3.4 | 3.5 | 3.3 | 3.5 | 3.5 |
| Zn1 – His116 |  | 2.0 | 2.0 | 2.2 | 1.9 | 2.1 | 2.0 |
| Zn1 – His118 |  | 2.0 | 2.0 | 2.1 | 2.1 | 2.0 | 2.1 |
| Zn1 – His196 |  | 2.0 | 2.0 | 2.1 | 1.9 | 2.0 | 2.2 |
| Zn2 – Asp120 |  | 2.0 | 2.1 | 2.5 | 1.9 | 2.1 | 2.2 |
| Zn2 – Cys221 |  | 2.3 | 2.4 | 1.9 | 2.0 | 2.2 | 2.3 |
| Zn2 – His263 |  | 2.1 | 2.1 | 2.7 | 2.2 | 2.2 | 1.9 |
| Zn1 – Zn1(IMP-27) |  | – | 0.3 | 0.5 | 0.2 | 0.9 | 0.5 |
| Zn2 – Zn2(IMP-27) |  | – | 0.8 | 0.9 | 0.3 | 0.7 | 0.9 |

^*^Protein Data Bank IDs are indicated in parentheses.

**Table S3. Kinetic parameters of the IMP-27 mutants.**

|  | wild-type |  |  |  | Y58F |  |  |  | T68P |  |  |  | K62N |  |  |
| --- | --- | --- | --- | --- | --- | --- | --- | --- | --- | --- | --- | --- | --- | --- | --- |
| Substrate  (concentration) | *k*_cat_  (s^-1^) | *K*_m_  (μM) | *k*_cat_/ *K*_m_  (s^-1^/μM) |  | *k*_cat_  (s^-1^) | *K*_m_  (μM) | *k*_cat_/ *K*_m_  (s^-1^/μM) |  | *k*_cat_  (s^-1^) | *K*_m_  (μM) | *k*_cat_/ *K*_m_  (s^-1^/μM) |  | *k*_cat_  (s^-1^) | *K*_m_  (μM) | *k*_cat_/ *K*_m_  (s^-1^/μM) |
| Ampicilin  (0.25 mM) | ND | ND | 0.0307 |  | ND | ND | 0.0201 |  | ND | ND | 0.0323 |  | ND | ND | 0.0201 |
| PenicillinG  (0.5 mM) | 27.2 ± 3.1 | 325 ± 50 | 0.0837 |  | ND | ND | 0.0550 |  | ND | ND | 0.0488 |  | ND | ND | 0.0428 |
| Cephalothin  (0.08 mM) | 618 ± 21 | 6.80 ± 0.55 | 91.2 |  | 629 ± 22 | 7.89 ± 0.96 | 80.4 |  | 400 ± 17 | 6.59 ± 0.58 | 60.8 |  | 526 ± 9 | 7.07 ± 0.29 | 74.5 |
| Meropenem  (0.06 mM) | 27.3 ± 1.9 | 6.38 ± 0.63 | 4.28 |  | 8.37 ± 0.33 | 5.57 ± 0.51 | 1.50 |  | 50.4 ± 2.4 | 19.3 ±3.2 | 2.61 |  | 7.25 ± 0.53 | 5.72 ± 0.92 | 1.27 |

|  | Q198P |  |  |  | H226Y |  |  |  | S261P |  |  |
| --- | --- | --- | --- | --- | --- | --- | --- | --- | --- | --- | --- |
| Substrate  (concentration) | *k*_cat_  (s^-1^) | *K*_m_  (μM) | *k*_cat_/ *K*_m_  (s^-1^/μM) |  | *k*_cat_  (s^-1^) | *K*_m_  (μM) | *k*_cat_/ *K*_m_  (s^-1^/μM) |  | *k*_cat_  (s^-1^) | *K*_m_  (μM) | *k*_cat_/ *K*_m_  (s^-1^/μM) |
| Ampicilin  (0.25 mM) | ND | ND | 0.0192 |  | 4.68 ± 0.00 | 173 ± 4 | 0.0271 |  | ND | ND | 0.0239 |
| PenicillinG  (0.5 mM) | ND | ND | 0.0454 |  | ND | ND | 0.0391 |  | ND | ND | 0.0584 |
| Cephalothin  (0.08 mM) | 584 ± 17 | 7.59 ± 0.60 | 77.2 |  | 593 ± 6 | 6.64 ± 0.70 | 89.9 |  | 366 ± 25 | 6.78 ± 0.20 | 53.9 |
| Meropenem  (0.06 mM) | 8.82 ± 0.21 | 5.89 ± 0.42 | 1.498 |  | 6.51 ± 0.09 | 4.52 ± 0.21 | 1.44 |  | 3.65 ± 0.12 | 3.03 ± 0.31 | 1.21 |

**TABLE S4** Kinetics measurement conditions

| Antibiotic | Concentration Range (μM) | Δε (M^-1^cm^-1^) | λ (nm) |
| --- | --- | --- | --- |
| Ampicillin | 250–500 | -900 | 235 |
| Benzylpenicillin | 250–750 | -775 | 233 |
| Cephalothin | 20–80 | -7660 | 262 |
| Cefotaxime | 40–80 | -7250 | 264 |
| Ceftazidime | 50–100 | -10300 | 265 |
| Cephalexin | 50–200 | -7800 | 266 |
| Meropenem | 100 | -6500 | 300 |


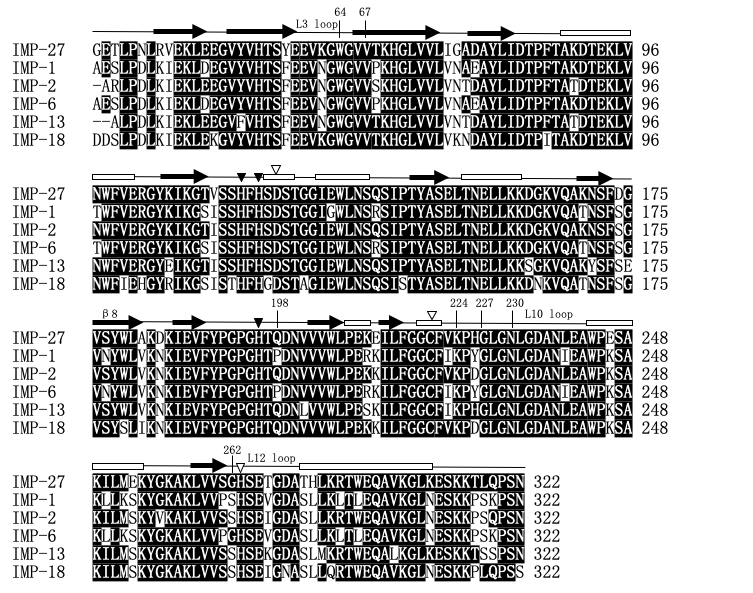


**FIG. S1.** Alignment of IMP-type enzymes with known structures. The amino acid sequences are as follows: IMP-1 (UniProt ID: P52699), IMP-2 (UniProt ID: Q6F3R2), IMP-6 (UniProt ID: K4PWX3), IMP-13 (UniProt ID: Q7WYA8), IMP-18 (UniProt ID: Q5U807), IMP-27 (UniProt ID: A0A286S0G7). Amino acid residues were numbered according to the standard numbering scheme for MBLs (9, 10). The residues were indicated by white letters on a black background when they were identical to the corresponding residues of IMP-27. Secondary structures are indicated above the sequence, with black arrows (β-strands) and white bars (α- helices). The residues coordinating to Zn1 are marked with filled triangles, and those coordinating to Zn2 are marked with open triangles. ClustalW was used to create the alignment (http://www.genome.jp/tools/clustalw/).


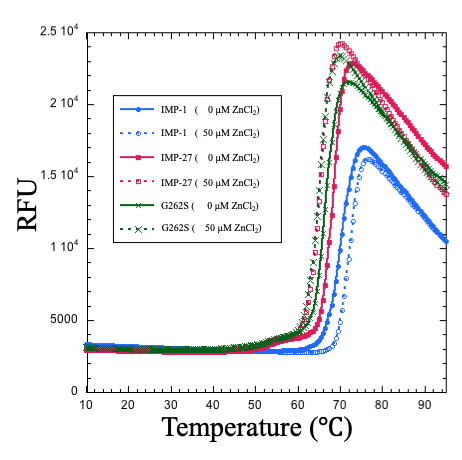


**FIG. S2.** Thermal stability of IMP-type enzymes. The relative fluorescence units (RFU) of SYPRO Orange measured as a function of temperature (°C). IMP-1 is shown in blue, IMP-27 in red, and the G262S mutant in green. Solid lines are the data measured in the absence of zinc ion, and dashed lines are the data measured in the presence of 50 μM ZnCl_2_. This figure was generated using KaleidaGraph ver.4.5.
